# Antimicrobial Resistance and Virulence Properties of *Campylobacter* Spp. Originating from Domestic Geese in Poland

**DOI:** 10.3390/ani10040742

**Published:** 2020-04-24

**Authors:** Beata Wysok, Joanna Wojtacka, Agnieszka Wiszniewska-Łaszczych, Joanna Szteyn

**Affiliations:** Department of Veterinary Public Health, Faculty of Veterinary Medicine, University of Warmia and Mazury in Olsztyn, Oczapowskiego 14, 10-917 Olsztyn, Poland; joanna.wojtacka@uwm.edu.pl (J.W.); aga@uwm.edu.pl (A.W.-Ł.); joanna.szteyn@uwm.edu.pl (J.S.)

**Keywords:** *Campylobacter*, geese, virulence genes, antimicrobial resistance

## Abstract

**Simple summary:**

*Campylobacter* spp. is the most common bacterial cause of human gastroenteritis in the world. Poultry and poultry products are considered to be the most important foodborne sources of campylobacteriosis in humans. In this study, the consumption of goose meat was shown as a potential risk for human campylobacteriosis. The common prevalence of these bacteria in geese cecum contributes to the high contamination of geese carcasses during slaughter. Moreover, *Campylobacter* isolated from geese harbored various virulence factors involved in adhesion and invasion and were resistant to some antimicrobial drugs.

**Abstract:**

A total of 240 samples were evaluated for the presence of *Campylobacter* spp. *Campylobacter* was found in 83.3% of the cecum contents samples and 52.5% of the neck skin samples from carcasses. The prevailing species was *C. jejuni*, accounting for 87.7% of all *Campylobacter* isolates, and the remaining 12.3% of isolates were *C. coli.* All *Campylobacter* isolates, independent of the sample origin and species, were positive for 6 out of 15 tested genes (*flaA*, *flhA*, *cadF*, *racR*, *ciaB*, and *cdtA* genes). The prevalence of *dnaJ*, *docA*, *pldA*, *cdtB*, *cdtC*, and *iam* genes was also very common (ranging from 86.5% to 98.8%). The lowest prevalence was noted for *virB11* and *wlaN* genes, both in *Campylobacter* isolates from cecum (12% and 19%) and carcasses (11.1% and 17.5%). None of the isolates tested, regardless of the sample origin, carried the *cgtB* gene. The highest resistance rates were observed for quinolones (90.8%) and tetracyclines (79.8%). Simultaneously, only single *Campylobacter* isolate was resistant to macrolides (0.6%) and none of the isolates showed resistance to aminoglycosides and amphenicols. The common presence of *Campylobacter* on geese carcasses as well as the detection of multidrug-resistant isolates indicate that consuming goose meat might cause a potential risk, therefore leading to human campylobacteriosis.

## 1. Introduction

Foodborne diseases are considered the most important health problem in the world [1]. They not only significantly affect human health and life but also have economic consequences for the individual, family, society, and the state [2]. The risk of foodborne diseases in humans increases with the increased ingestion of animal origin products. In recent years, both in Poland and throughout the European Union, the structure of meat consumption has changed as poultry is increasingly replacing red meat. Although chickens and turkeys dominate the world poultry industry, geese and duck products are also willingly chosen by consumers not only because of their taste but also their nutritional values. The leading goose producer in the world is China with 94.1% of global production. Poland is ranked second, followed by Hungary and Egypt, with the production of approximately 28,261 tons, which accounted for 1.2% of the world production in 2018 (www.faostat.fao.org). Geese production in Poland is seasonal and the breeding cycle begins in spring and ends in autumn. Poultry meat has been implicated in many foodborne disease outbreaks throughout the world [3,4,5]. Despite the rapid and highly automated slaughter of poultry, the risk of contamination and the spread of bacteria during slaughter is considerable [6]. It is believed that the source of bacterial contamination in poultry meat is essentially the intestine or gut content, which may come into contact with carcasses during slaughter, either directly or indirectly [7]. In recent years, much attention has been paid to the importance of poultry in causing infections caused by bacteria belonging to the genus *Campylobacter*. This microorganism is currently considered to be the most common bacterial cause of human gastroenteritis [8]. 

Campylobacteriosis is a zoonotic disease and human infection occurs as a result of ingesting live cells of these bacteria with food. Black et al. [9], in experimental studies on *Campylobacter* infection in human volunteers, estimated the infective dose is as low as 800 cfu. However, Hara-Kudo and Takatori [10], in recent studies, estimated that the dose of *C. jejuni* required for the development of campylobacteriosis can be as low as 360 cfu. Therefore, people are infected relatively easily and often. Humans with *Campylobacter* infection experience water or bloody diarrhea, abdominal cramps, nausea, and fever [11]. The onset of symptoms usually occurs 24 to 72 h following ingestion and may last up to 7 days [8]. In most cases, *Campylobacter* enteritis is usually self-limiting; however, complications may occur in some persons. Approximately 1 in 1000 infected individuals develops Guillain–Barré syndrome (GBS), a serious autoimmune-mediated neurological disorder that can cause symptoms ranging from weakness of extremities to complete paralysis and respiratory insufficiency [12]. 

The pathogenesis of *Campylobacter* infection is complex and still poorly understood [13]. Recently, some genes have been recognized as being responsible for the expression of pathogenicity, i.e., determining flagella-mediated motility (*flaA* and *flhA*), adherence abilities to intestinal epithelial cells (*cadF*, *dnaJ*, *racR*), invasion abilities to the host cells (*ciaB*, *iam*, *virB11*), cytotoxin production (*cdtA*, *cdtB* and *cdtC*), and responsible for the expression of Guillain–Barré syndrome (*wlaN* and *cgtB*) [14,15,16,17,18].

Most cases of human campylobacteriosis are self-limiting and do not require special therapy, except fluid and electrolyte supplementation. However, in the case of severe and prolonged diarrhea and the presence of *Campylobacter* in the blood, particularly in the young, elderly, and in individuals with compromised immunity, antibiotic treatment is recommended [19]. The most common antimicrobial agents used in the treatment of *Campylobacter* infections are macrolides and fluoroquinolones, while in the case of systemic infection, tetracyclines and gentamicin are used [20]. However, the broad use of these chemotherapeutics both in human medicine and animal production contributes to the increasing number of *Campylobacter* isolates resistant to clinically important antibiotics and this rising resistance is a concern for public health [19]. 

The aim of the study was to investigate and determine whether domestic geese in Poland are contaminated with *Campylobacter* spp. and may be a potential source of infection in humans. Moreover, isolated *Campylobacter* spp. were tested for virulence-associated markers involved in motility, adhesion, invasion, cytotoxin production, and the development of Guillain–Barré syndrome, as well as resistance to clinically relevant antibiotics.

## 2. Material and Methods

### 2.1. Isolation of Bacterial Strains

A total of 240 samples were evaluated for the presence of *Campylobacter* spp. The samples were taken in two slaughterhouses in the north-east of Poland. Because of the seasonal slaughter of geese, the samples were taken from October to December 2016, and October to December 2017. Overall, 24 flocks were tested on separate occasions. Five geese were randomly selected after evisceration and chilling from every flock. Cecum was dissected after evisceration from each bird. After chilling the carcasses to 4 °C, 10 g of skin sample from the neck of each goose was taken with a sterile blade. All the samples were transported to the laboratory at 2–4 °C. The laboratory analysis was conducted in accordance with ISO 10272-1:2017 [21]. In the laboratory, each cecum was opened aseptically, and a content of intestine was taken. The amount of 1 g of intestinal content was supplemented with 9 mL of Bolton enrichment broth (Oxoid, Hampshire, UK). Skin samples of 10 g were taken and submerged in 90 mL of Bolton enrichment broth. All samples were homogenized in stomacher and incubated for 4 h at 37 °C and subsequently for 44 h at 41.5 °C under microaerobic conditions (5% O_2_, 10% CO_2_, and 85% N_2_). The cultures were then plated onto mCCDA (charcoal cefoperazone deoxycholate modified agar, Oxoid) and Karmali agar (Oxoid). All the plates were incubated for 24–48 h at 41.5 °C microaerobically as described above. The plates were examined for morphologically typical *Campylobacter* colonies, which were confirmed by microscopic morphology, motility, microaerobic growth at 25 °C, and the presence of oxidase. The isolates were subcultured only once in order to minimize cultural changes and then stored at −80 °C in defibrinated horse blood (Oxoid) with the addition of glycerol (80:20 v/v).

### 2.2. Species Identification

All *Campylobacter* isolates were identified by the PCR method based on the amplification of genus-specific 16S rRNA gene, the *mapA* gene specific for *C. jejuni* and the *ceuE* gene specific for *C. coli*. All primers used in the study are shown in Table 1. *Campylobacter* isolates cultured on Columbia agar medium with blood (Oxoid) were suspended in 1 mL of sterile water, and centrifuged at 13 000x g for 1 min. The precipitate was suspended in Tris buffer. DNA isolation was performed using the Genomic-Mini Kit (A&A Biotechnology, Gdynia, Poland) according to the manufacturer’s instructions. The purity and concentration of the DNA obtained were determined spectrophotometrically and after appropriate dilution was used in the PCR assay. Amplification was performed in a reaction mixture containing 5 μL of the PCR buffer (10 times concentrated), 5 μL of dNTPs (final concentration of 200 µM), 0.5 μL of each primer (final concentration 0.1 µM), 10 μL MgCl_2_ (final concentration of 5 mM), 2 μL (2 U) of thermostable Taq polymerase (Thermo Fisher Scientific, Waltham, MA, USA), 5 μL of template DNA, and DNase- and RNase-free deionized water to a final volume of 50 μL. All PCR reactions were carried out using the following conditions: Initial denaturation at 94 °C for 5 min followed by 30 cycles of denaturation for 1 min at 95 °C, annealing at a temperature specific to the primer pair for 1 min, and extension for 1 min at 72 °C. The final elongation step was carried out at 72 °C for 5 min. A positive control consisting of DNA extracted from *C. jejuni* ATCC 33291 and *C. coli* ATCC 43478 as well as a negative PCR control consisting of PCR-grade water were included in each PCR run. The PCR product was identified on a 2% agarose gel stained with ethidium bromide at a concentration of 5 μg/mL. The sizes of the amplification products obtained were compared with the 100-bp molecular weight marker.

### 2.3. Virulence Factor Genes

The confirmation of the presence of genes involved in motility (*flaA* and *flhA*), adhesion (*cadF*, *dnaJ*, *racR, docA*), invasion (*pldA*, *virB11*, *iam*, *ciaB*), cytotoxin production (*cdtA*, *cdtB*, and *cdtC*), and GB syndrome (*wlaN* and *cgtB*) were undertaken. The PCR mixture and amplification of virulence genes were carried out as described previously [27]. All primers used in the study are shown in Table 1.

### 2.4. Antimicrobial Resistance

Antimicrobial resistance was examined by the diffusion-disk method according to the protocol of the European Committee on Antimicrobial Susceptibility Testing (EUCAST) for fastidious organisms. All *Campylobacter* isolates were suspended in brain heart infusion (BHI) broth to a turbidity equivalent to a 0.5 McFarland standard. Mueller-Hinton agar plates supplemented with 5% of defibrinated horse blood (Oxoid) and 20mg/L of β-Nicotinamide Adenine Dinucleotide (β – NAD) (Sigma Aldrich, St. Louis, MO, USA) were inoculated with the suspension prepared. The following antibiotic disks were placed on the surface of dry plates: Erythromycin (ERY, 15 µg), gentamicin (G, 10 µg), ciprofloxacin (CIP, 5 µg), ampicillin (AMP, 10 µg), tetracycline (TET, 30 µg), chloramphenicol (CHL, 30 µg), and nalidixic acid (NAL, 30 µg). The plates were incubated at 41 ± 1 °C for 24–48 h in a microaerophilic atmosphere. Zones of inhibited growth for erythromycin, ciprofloxacin, and tetracycline were determined according to EUCAST breakpoints (www.eucast.org/clinical_breakpoints), and The Clinical & Laboratory Standards Institute (CLSI, guideline M45-A3) breakpoints were used for the remaining tested antibiotics [28]. The results were interpreted as resistant or sensitive. The inhibition zone readings defined as intermediate were classified as resistant. The strains that showed resistance to three or more classes of antimicrobial agents were considered as multidrug resistant (MDR).

### 2.5. Statistical Analysis

Statistical tests were performed using Statistica (StatSoft, version 13.3, Poland). The chi-square test was used to determine differences in the prevalence of virulence marker genes and antimicrobial resistance of *Campylobacter* isolated from cecum and carcasses. For small sample sizes, Yates’ correction was also used. Statistical significance was defined as *p* < 0.05.

## 3. Results

### 3.1. Isolation and Identification of Bacterial Strains

In the study, a total of 240 samples from geese were analyzed for the presence of *Campylobacter* spp. A total of 100 (83.3%) *Campylobacter* isolates were obtained from 120 samples of cecum contents. Of the 120 samples of neck skin from carcasses, 63 (52.5%) were found to be positive. Out of 24 flocks examined, 20 (83.3%) showed a prevalence of *Campylobacter* spp. in 5/5 (100%) cecum samples tested. In the remaining four flocks (16.7%), *Campylobacter* was not noted in any cecum tested (Table 2). Carcass contamination was noted in 17 out of 20 (70.8%) flocks with a confirmed prevalence of *Campylobacter* spp. in cecum samples. There were 9 flocks showing 5/5 (100%), 8 flocks showing 2/5 (40%) contamination of the carcasses, and 3 flocks without contamination of carcasses. Out of four flocks with cecum negative, *Campylobacter* spp. was recovered from one carcass.

PCR analysis, regardless of the sample origin, showed that the majority (87.7%) of *Campylobacter* isolates were identified as *C. jejuni*. The remaining 12.3% were identified as *C. coli*. 

### 3.2. Virulence Factor Genes

**Figure 1:**
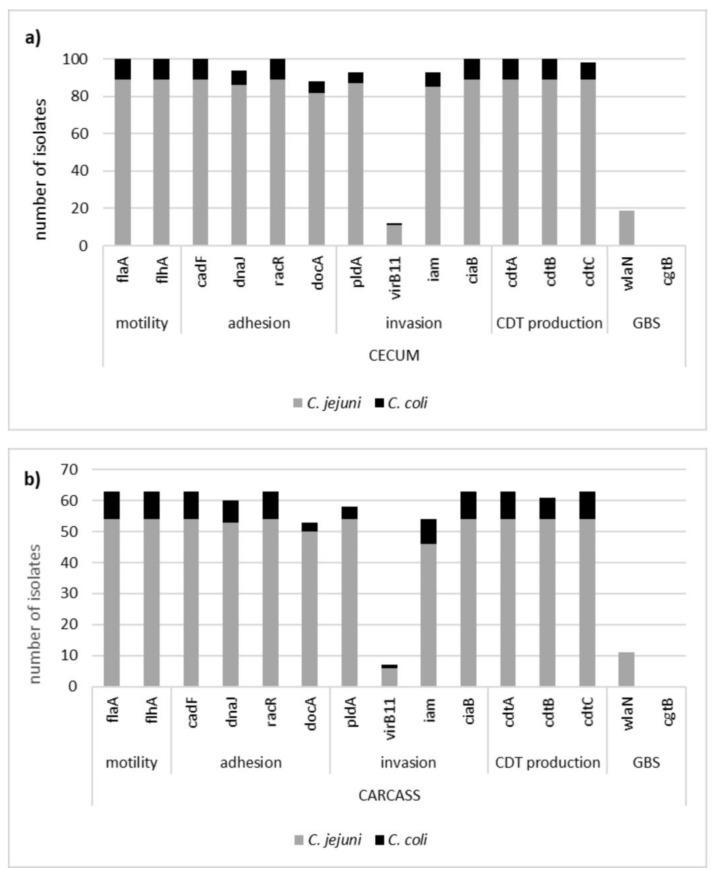
Prevalence of virulence markers in *Campylobacter* isolates originating from geese cecum (**a**) and carcasses (**b**).

A total of 163 *Campylobacter* isolates, including 100 isolates from intestinal contents and 63 isolates from geese carcasses, were screened for the prevalence of virulence markers (Figure 1). 

All *Campylobacter* isolates, regardless of the sample origin and species, were positive for 6 out of 15 tested genes (*flaA*, *flhA*, *cadF*, *racR*, *ciaB*, and *cdtA*). In the case of *Campylobacter* isolates obtained from geese cecum, high prevalence rates were also observed for the *dnaJ* and *docA* genes involved in adhesion (94% and 88%), for the *pldA* gene associated with invasion (93%), and the *cdtB* and *cdtC* genes associated with toxin production (both at 98%). Similar rates were observed in *Campylobacter* isolates from carcasses, and 95.2%, 84.1%, 92.1%, 96.8%, 100%, and 85.7% of isolates were positive for *dnaJ*, *docA*, *pldA*, *cdtB*, *cdtC,* and *iam* genes. The lowest prevalence was noted for the *virB11* and *wlaN* genes, both in *Campylobacter* isolates from cecum (12% and 19%) and carcasses (11.1% and 17.5%). None of the isolates tested, regardless of the sample origin, carried the *cgtB* gene.

### 3.3. Antimicrobial Resistance

Overall, the highest resistance rates were observed for quinolones (90.8%) and tetracyclines (79.8%). Simultaneously, only a single *Campylobacter* isolate was resistant to macrolides (0.6%) and no isolate showed resistance to aminoglycosides and amphenicols. 

None of the *Campylobacter* isolates originating from cecum contents were resistant to gentamicin and chloramphenicol and only a single isolate of *C. coli* was resistant to erythromycin (1%). The resistance rate to ampicillin was 32%, while the frequency of resistance to ciprofloxacin, nalidixic acid, and tetracycline was found to be high at the level of 92%, 88%, and 81%, respectively (Table 3).

Regarding *Campylobacter* isolates obtained from geese carcasses, the highest resistance was observed for ciprofloxacin (93.6%), nalidixic acid (90.5%), and tetracycline (77.8%). Simultaneously, all isolates were sensitive to gentamicin, erythromycin, and chloramphenicol. A low number of isolates were resistant to ampicillin (36.5%) (Table 3). 

Eleven different antimicrobial-resistant patterns were noted among *Campylobacter* isolates (Table 4). Only 5 out of 163 (3.1%) of the isolates obtained from geese were found to be susceptible to all antimicrobials tested, including 3/100 (3%) and 2/63 (3.2%) isolates from cecum and carcasses, respectively. Multidrug resistance to at least three different antimicrobial classes was found among *Campylobacter* spp. isolates both from cecum (28%) and carcass samples (30.2%).

## 4. Discussion

Comparing the results of the current study regarding the degree of the contamination of geese carcasses by *Campylobacter* spp. and determining the virulence properties of the isolates obtained with the results of other authors was found to be difficult due to the limited literature on this subject. It has been well documented that poultry meat is frequently involved in human campylobacteriosis, accounting for 20% to 30% of cases [29]. The common prevalence of *Campylobacter* both in raw chicken and turkey samples has been demonstrated in different geographical regions at the level of 50.2% and 41.1% in Poland [30], 61.7% and 36% in Iran [31], and 49.9% and 37.5% in Ireland [32]. However, the studies performed by Kim et al. [33] and Little et al. [34] underlined that duck meat is also an important source that can transmit *Campylobacter*, noting that 62.3% and 50.7% of samples were contaminated by this pathogen. Simultaneously, our study demonstrated that geese can be implicated in human campylobacteriosis as overall, 83.3% and 52.5% of the cecum and carcasses samples were positive for *Campylobacter* spp. The prevailing species, regardless of the sample origin, was *C. jejuni* (87.7%), whereas 12.3% of isolates were *C. coli*. These findings are consistent with the opinion that *C. jejuni* is more prevalent in poultry, whereas *C. coli* is more common in pigs and both can contaminate cattle [35,36]. Additionally, in humans, *C. coli* is less prevalent than *C. jejuni* [8,37].

During slaughter operations, the microorganisms are spread over the entire carcass. In this study, a correlation between the prevalence of *Campylobacter* spp. in the digestive tract of geese and the contamination of carcasses was confirmed. After the slaughter of 20 (85%) flocks with a confirmed prevalence of *Campylobacter* spp. in cecum samples, the contamination of geese carcasses was noted in 17 flocks (85%). Moreover, with high numbers of birds slaughtered per hour, it is not possible to conduct washing and disinfection of all parts of the machines between the slaughtering of individual flocks, as a consequence of which the total elimination of cross contamination on the slaughter line is impossible. The above situation was confirmed in our study. Contamination was detected in carcasses originating from geese flocks initially free from *Campylobacter* spp. These findings are in line with the results obtained by Hiett et al. [38], suggesting that the external environment may contribute to *Campylobacter* contamination during poultry production and processing.

In this study, the prevalence of 15 genes involved in motility, adhesion, invasion, production of cytotoxin, and GBS were examined to determine the pathogenic properties of *Campylobacter* isolates obtained from geese. Colonization of the mucous lining of the gastrointestinal tract is the first step of *Campylobacter* infection, and the *Campylobacter* flagellar filament composed of two homologous flagellins: FlaA and FlaB, encoded by adjacent genes, appear essential in this process [39]. FlhA is a key component of the flagellar export apparatus, and inactivation of the *flhA* gene leads to the loss of FlaA expression and motility but also to autoagglutination and invasion [34]. We noted that all *Campylobacter* isolates were positive for the *flaA* and *flhA* genes, regardless of the species and sample origin. These findings are in accordance with data presented previously in Poland [13], Vietnam [40], Brazil [41], and Denmark [42]. Besides, the above authors reported on *Campylobacter* isolates obtained from chicken or turkey. Moreover, the flagellum plays a role not only in motility, but it can also secrete molecules that promote *Campylobacter* adhesion to and invasion into host cells [43,44]. Among the factors related to adhesion, a 37-kDa outer membrane protein CadF, encoded by the *cadF* gene, affects the binding of *Campylobacter* to host fibronectin [45,46]. The results presented by numerous authors underline the common prevalence of this marker in *Campylobacter* isolates obtained from different sources [13,15,40]. Similar observations were noted in our study. All isolates originating from cecum and carcasses were positive for the *cadF* gene. Additionally, other adhesins (encoded by the *dnaJ*, *racR*, and *docA* genes) have been identified as important for *Campylobacter* adherence in vitro and colonization in vivo [47,48]. In the current study, all *Campylobacter* isolates, both from cecum and carcasses, were positive for the *racR* gene. The remaining two examined genes involved in adhesion (*dnaJ* and *docA*) were found in 94% and 88% of the *Campylobacter* isolates from cecum and in 95.2% and 84.1% of isolates from carcasses, respectively. The *dnaJ*, *racR*, and *docA* genes were commonly distributed in *Campylobacter* strains in different geographic regions. Frazao et al. [41] showed the prevalence of these genes in all isolates from poultry feces and carcasses in Brazil. Additionally, Wieczorek et al. [49] showed the high prevalence of the *docA* and *racR* genes in 100% *Campylobacter* strains isolated from chicken feces and in 98.1% and 95.5% of the isolates from poultry carcasses, respectively.

Many virulence factors have been associated with *Campylobacter* invasion to epithelial cells, including the *pldA*, *virB11*, *iam*, and *ciaB* genes. In our study, a significantly lower rate of prevalence was marked for the *virB11* gene that was noted at the level of 12% in isolates from cecum and 11.1% in isolates from carcasses when compared to the prevalence of the *ciaB*, *pldA*, and *iam* genes (noted in almost all isolates, regardless of the sample origin). The low prevalence of the *virB11* gene has been previously noted in Poland among *Campylobacter* isolates originating from chicken feces (0.6%) and carcasses (2.5%) [13]. Additionally, Kim et al. [33], in studies on *Campylobacter* strains isolated from chicken and duck meat, detected a low rate of this marker, respectively, in 7.8% and 6.7% of the isolates. This marker was also identified sporadically in human clinical isolates, and the *virB11* mutant can cause significantly less severe symptoms in vivo [48,49,50]. On the other hand, the rate of other markers involved in invasion, including the *iam* (invasion-associated marker), *ciaB* (*Campylobacter* invasive antigen B), and *pldA* (encoding a phospholipase A) genes, were commonly reported in previously conducted studies. The *pldA* and *ciaB* genes have been noted in *Campylobacter* strains isolated from chicken carcasses at the rate of 63.6% and 67.3% in Brazil [51], as well as in South Korea [33] in chicken isolates (94.4% and 95.6%) and duck isolates (91.1% and 88.9%). These findings are in accordance with our data. The *ciaB* gene was found in 100% of the *Campylobacter* isolates regardless of the sample origin and the *pldA* gene in 93% and 92.1% of the isolates from feces and carcasses. Dissimilar results were observed regarding the *iam* gene. In this study, the *iam* marker was noted in 93% of the isolates from feces and in 85.7% of the isolates from carcasses. Similar results were observed by Kim et al. [33], noting this gene in 97.8% of chicken and 88.9% of duck-origin *Campylobacter* isolates. In contrast to our result, a significantly lower rate of the *iam* gene was noted in Poland by Wieczorek et al. [49] at the level of 26.2% in isolates from poultry feces and 8.9% in isolates from carcasses. 

The ability of *Campylobacter* strains to produce toxins also plays a significant role in the course of *Campylobacter* infection. The cytolethal distending toxin (CDT) composed of three subunits CdtA, CdtB, and CdtC is the best characterized *Campylobacter* toxin [52]. In this study, overall, 97.5% of *Campylobacter* isolates possessed three tested *cdt* genes and the rate was slightly higher in *C. jejuni* (100%) than in *C. coli* (80%) isolates. Previous studies conducted on isolates from chicken carcasses by Datta et al. [15] in Japan and Rozynek et al. [53] in Poland reported that the prevalence of each of the *cdtA*, *cdtB*, or *cdtC* genes in *C. jejuni* isolated from poultry exceeded 80%.

Most *Campylobacter* infections are self-limited; however, complications may also occur. *Campylobacter* enteritis was described as the predominant bacterial infection preceding the Guillain–Barré syndrome (GBS) [54]. The *Campylobacter* strains that can elicit GBS carry either the *wlaN* or *cgtB*, genes involved in LOS (sialylated lipooligosaccharide) synthesis [18]. In the current study, 19% of the *Campylobacter* isolates from intestinal contents and 17.5% of the isolates from carcasses were positive for the *wlaN* gene. Similar rates were obtained previously by Wieczorek et al. [49] in Poland at the level of 13.7% and 17.2% in isolates from chicken feces and carcasses, and by Guirado et al. [18] in Spain at the level of 22% in chicken isolates. Additionally, in human origin *Campylobacter* isolates, the presence of the *wlaN* gene was estimated to be between 20% in Spain [18] and 17.4% in Poland [49]. Although several reports indicated that *cgtB* and *wlaN* may coexist, the studies performed by Guirado et al. [18] suggested the contrary, since none of the isolates tested carried both genes. In this study, none of the isolates examined were positive for the *cgtB* gene. Generally, this gene is detected in the most strongly invasive strains and rarely in non-invasive strains [23]. 

Antimicrobial agents are used widely in human and veterinary medicine. The transmission of resistance from animals to humans can take place through a variety of routes, though the foodborne route is probably the most significant [55]. In cases of campylobacteriosis with a course of systemic infections, infections in immune-suppressed patients, and severe or long-lasting infections, when antibiotic treatment is needed, macrolides are often recommended as the drug of first choice [56]. The results obtained showed a very low resistance rate to erythromycin (0.6% of resistant isolates in total), noting only one resistant *C. coli* strain originating from cecum. In previous studies in Poland, the levels of resistance to erythromycin among *Campylobacter* isolated from poultry were low, ranging from 0% [57] to single isolates resistant to this drug (3% of *C. coli* and 1.7% of *C. jejuni* isolates) [58]. Similar findings were noted by Bywater et al. [59], showing a lack of resistance to erythromycin in all *Campylobacter* strains isolated from poultry in abattoirs in France, Germany, the Netherlands, and Sweden. 

Quinolones and tetracyclines are recommended as alternative drugs in the treatment of *Campylobacter* infection [56]. Unfortunately, in the present study, very high resistance levels were noted for these antimicrobials, showing 92.6% of *Campylobacter* isolates as resistant to ciprofloxacin, 88.9% to nalidixic acid, and 79.8% to tetracycline. According to Unicomb et al. [60], the spread of quinolone resistance in *Campylobacter* isolates might have originated from the excessive use of veterinary quinolones (e.g., enrofloxacin or danofloxacin) in food-producing animals. The confirmation of this argument may be found in studies conducted in Australia, where the use of quinolones is banned in food-producing animals and a very low rate of resistance at the level of 0–2% was reported [61]. Additionally, the common use of tetracyclines both in the therapy of human and animal infections is implicated in the increased number of isolates resistant to this antimicrobial agent. High rates of resistance to quinolones and tetracyclines have also been reported previously in *Campylobacter* isolates from chicken feces and carcasses, as from duck and turkey isolates [33,49,58]. 

Ampicillin, like tetracycline, shows activity against *Campylobacter*, but in general, both are not recommended for the treatment of campylobacteriosis because the rates of resistance of these antimicrobials are too high to be useful [62]. The results obtained by these authors showed that 65% of *Campylobacter* isolates from broiler slaughterhouses in southern Brazil were resistant to β-lactams. Similarly, in Turkey, 67% of goose isolates were ampicillin resistant [63]. In turn, the current study revealed a slightly lower resistance rate, ranging from 32% among *Campylobacter* isolates originating from cecum to 36.5% among isolates originating from geese carcasses. 

Chloramphenicol and gentamicin resistance in *Campylobacter* spp. have been reported to be low [64]. It has been confirmed by the results of this research that all isolates both from geese cecum and carcasses are susceptible to these antimicrobial drugs. Similarly, none of the duck and goose isolates originating from Malaysia [65], chicken isolates originating from Canada [66], and turkey isolates from Germany [67] were resistant to chloramphenicol and gentamicin.

The emergence of multidrug resistance to at least three antimicrobial classes was underlined in this study. We found a slightly higher number of *Campylobacter* isolates resistant to at least three antimicrobial classes among the isolates originating from carcasses samples (30.2%) compared to the isolates from cecum samples (28%). The majority of MDR isolates were resistant to quinolones, tetracyclines, and β-lactams. Only one *C. coli* isolate obtained from cecum was resistant to quinolones, macrolides, and tetracyclines. A slightly lower rate of multidrug-resistant *Campylobacter* isolates was reported by Wieczorek et al. [68], who noted a level of 20.6% of the poultry isolates tested during 2014–2018 in Poland. However, these isolates were mainly resistant to quinolones, aminoglycoside, and tetracyclines. Higher multidrug resistance levels ranging from 60.2% up to 100% were noted among *Campylobacter* isolates from geese and ducks in Malaysia [65] and among chicken isolates in Africa [69].

## 5. Conclusions

The common prevalence of *Campylobacter* spp. in the intestinal tract of geese leads to the contamination of their carcasses during slaughter and processing. Simultaneously, cross contamination of carcasses originating from *Campylobacter*-negative flocks, occurring during slaughter, is considered to be the main hygienic problem in slaughterhouses. Moreover, the prevalence of virulence markers involved in motility, adhesion, invasion, and cytotoxin production were common among *Campylobacter* isolates originating from geese. Macrolides, aminoglycosides, and amphenicol should be considered as the drugs of choice in *Campylobacter* infection treatment. Other antibiotics, such as quinolones, tetracyclines, and β-lactams, should not be taken into consideration as alternative drugs for the treatment of campylobacteriosis, due to high rates of resistance. Thus, the common presence of *Campylobacter* on geese carcasses as well as the detection of multidrug-resistant isolates, indicate that the consumption of goose meat might cause the potential risk of human campylobacteriosis.

## Figures and Tables

**Table 1:** PCR primers used in the study.

Target Gene	Sequences (5’–3’)	Product Size (bp)	Annealing Temperature °C	References
*16S rRNA* for *Campylobacter* spp.	F-ATCTAATGGCTTAACCATTAAAC R-GGACGGTAACTAGTTTAGTATT	857	58	[22]
*mapA* for *C. jejuni*	F-CTATTTTATTTTTGAGTGCTTGTG R-GCTTTATTTGCCATTTGTTTTATTA	589	58	[22]
*ceuE* for *C. coli*	F-AATTGAAAATTGCTCCAACTATG R-TGATTTTATTATTTGTAGCAGCG	462	58	[22]
*flaA*	F-AATAAAAATGCTGATAAAACAGGTG R-TACCGAACCAATGTCTGCTCTGATT	855	53	[15]
*flhA*	F-GGAAGCGGCACTTGGTTTGC R-GCTGTGAGTGAGATTATAGCAG	735	53	[23]
*dnaJ*	F-ATTGATTTTGCTGCGGGTAG R-ATCCGCAAAAGCTTCAAAAA	177	50	[24]
*cadF*	F-TTGAAGGTAATTTAGATATG R-CTAATACCTAAAGTTGAAAC	400	45	[25]
*virB11*	F-TCTTGTGAGTTGCCTTACCCCTTTT R-CCTGCGTGTCCTGTGTTATTTACCC	494	53	[15]
*docA*	F-ATAAGGTGCGGTTTTGGC R-GTCTTTGCAGTAGATATG	725	50	[23]
*Iam*	F-GCGCAAAATATTATCACCC R-TTCACGACTACTATGCGG	518	52	[26]
*ciaB*	F-TGCGAGATTTTTCGAGAATG R-TGCCCGCCTTAGAACTTACA	527	54	[24]
*racR*	F-GATGATCCTGACTTTG R-TCTCCTATTTTTACCC	584	45	[15]
*pldA*	F-AAGCTTATGCGTTTTT R-TATAAGGCTTTCTCCA	913	45	[15]
*cdtA*	F-CCTTGTGATGCAAGCAATC R-ACACTCCATTTGCTTTCTG	370	49	[15]
*cdtB*	F-CAGAAAGCAAATGGAGTGTT R-AGCTAAAAGCGGTGGAGTAT	620	51	[15]
*cdtC*	F-CGATGAGTTAAAACAAAAAGATA R-TTGGCATTATAGAAAATACAGTT	182	47	[15]
*wlaN*	F-TGCTGGGTATACAAAGGTTGTG R-ATTTTGGATATGGGTGGGG	330	55	[23]
*cgtB*	F-TAAGAGCAAGATATGAAGGTG R-GCACATAGAGAACGCTACAA	561	52	[14]

**Table 2:** *Campylobacter* isolation rate in geese ceca and carcasses samples per farm.

Flocks	Sampling	No. of Slaughtered Geese	No. of Positive Samples/No. of Tested Samples			
			Ceca		Carcasses	
			n	%	n	%
A	X 2016	1470	5/5	100	5/5	100
B	X 2016	5100	5/5	100	2/5	40.0
C	X 2016	3500	5/5	100	5/5	100
D	X 2016	6170	-	-	-	-
E	X 2016	750	5/5	100	5/5	100
F	XI 2016	1500	5/5	100	5/5	100
G	XI 2016	2300	5/5	100	2/5	40.0
H	XI 2016	1760	-	-	-	-
I	XI 2016	800	5/5	100	5/5	100
J	XI 2016	1230	5/5	100	2/5	40.0
K	XII 2016	2100	5/5	100	2/5	40.0
L	XII 2016	2270	5/5	100	-	-
M	X 2017	4900	5/5	100	2/5	40.0
N	X 2017	540	5/5	100	5/5	100
O	X 2017	2200	5/5	100	2/5	40.0
P	X 2017	7900	-	-	-	-
Q	XI 2017	3080	-	-	1/5	20.0
R	XI 2017	1890	5/5	100	-	-
S	XI 2017	1550	5/5	100	5/5	100
T	XI 2017	4300	5/5	100	2/5	40.0
U	XI 2017	2250	5/5	100	5/5	100
V	XII 2017	1800	5/5	100	-	-
W	XII 2017	11000	5/5	100	2/5	40.0
Z	XII 2017	2200	5/5	100	5/5	100

**Table 3:** Antimicrobial resistance of *Campylobacter* isolates originating from geese cecum and carcasses.

Antimicrobials	No. of Resistant Isolates (%)								
	Cecum			Carcass			Together		
	*C. jejuni* n = 89	*C. coli* n = 11	Total n = 100	*C. jejuni* *n = 54*	*C. coli* n = 9	Total n = 63	*C. jejuni* n = 143	*C. coli* n = 20	Total n = 163
Ciprofloxacin (CIP)	82 (92.1)	10 (90.9)	92 (92.0)	50 (92.6)	9 (100)	59 (93.6)	132 (92.3)	19 (95.0)	151 (92.6)
Nalidixic acid (NAL)	79 (88.7)	9 (81.8)	88 (88.0)	49 (90.7)	8 (88.9)	57 (90.5)	128 (89.5)	17 (85.0)	145 (88.9)
Gentamicin (G)	0 (0.0)	0 (0.0)	0 (0.0)	0 (0.0)	0 (0.0)	0 (0.0)	0 (0.0)	0 (0.0)	0 (0.0)
Erythromycin (ERY)	0 (0.0)	1 (9.0)	1 (1.0)	0 (0.0)	0 (0.0)	0 (0.0)	0 (0.0)	1 (5.0)	1 (0.6)
Tetracycline (TET)	72 (80.9)	9 (81.8)	81 (81.0)	42 (77.8)	7 (77.8)	49 (77.8)	114 (79.7)	16 (80.0)	130 (79.8)
Ampicillin (AMP)	23 (32.6)	3 (27.3)	32 (32.0)	19 (35.2)	4 (44.4)	23 (36.5)	48 (33.6)	7 (35.0)	55 (33.7)
Chloramphenicol (CHL)	0 (0.0)	0 (0.0)	0 (0.0)	0 (0.0)	0 (0.0)	0 (0.0)	0 (0.0)	0 (0.0)	0 (0.0)

**Table 4:** Distribution of the multiple antimicrobial resistance profile in *Campylobacter* strains from geese cecum and carcasses.

Source and Species of Isolates	Gene Pattern	No. of Isolates (%)
Isolates from Cecum		
*C. coli*	CIP_NAL	1/11 (9.1)
	CIP_NAL_TET	5/11 (45.5)
	CIP_NAL_TET_AMP *	3/11 (2.3)
	CIP_NAL_ERY_TET *	1/11 (9.1)
*C. jejuni*	TET	3/89 (3.4)
	TET_AMP	2/89 (2.2)
	CIP_NAL	11/89 (12.4)
	CIP_AMP	1/89 (1.1)
	CIP_NAL_TET	43/89 (48.3)
	CIP_NAL_AMP	3/89 (3.4)
	CIP_TET_AMP *	2/89 (2.2)
	CIP_NAL_TET_AMP *	22/89 (24.7)
**Isolates from Carcasses**		
*C. coli*	CIP_NAL	1/9 (11.1)
	CIP_NAL_TET	4/9 (44.4)
	CIP_NAL_AMP	1/9 (11.1)
	CIP_TET_AMP *	1/9 (11.1)
	CIP_NAL_TET_AMP *	2/9 (22.2)
*C. jejuni*	CIP	1/54 (1.8)
	TET	1/54 (1.8)
	TET_AMP	1/54 (1.8)
	CIP_NAL	7/54 (12.9)
	CIP_NAL_AMP	2/54 (3.7)
	CIP_NAL_TET	24/54 (44.4)
	CIP_NAL_TET_AMP *	16/54 (29.6)

* indicate resistance of *Campylobacter* isolates to at least three classes of the antimicrobial agents.
